# Current management of gastro-oesophageal reflux disease—treatment costs, safety profile, and effectiveness: a narrative review

**DOI:** 10.1093/gastro/goad008

**Published:** 2023-04-18

**Authors:** Tahmina Lata, Jodie Trautman, Philip Townend, Robert B Wilson

**Affiliations:** Faculty of Medicine and Health, University of Sydney, Camperdown, NSW, Australia; Upper Gastrointestinal Surgery Department, University of New South Wales, Liverpool Hospital, Liverpool, NSW, Australia; General Surgery Department, Wollongong Hospital, Wollongong, NSW, Australia; General Surgery Department, Gold Coast University Hospital, Southport, QLD, Australia; Upper Gastrointestinal Surgery Department, University of New South Wales, Liverpool Hospital, Liverpool, NSW, Australia

## Abstract

**Background:**

The purpose of this study was to review the current management of gastro-oesophageal reflux disease (GORD), including treatment costs, safety profile and effectiveness.

**Methods:**

A literature review was performed of randomized-controlled trials, systematic reviews, Cochrane reports and National/Societal guidelines of the medical, endoscopic and surgical management of GORD. Proton pump inhibitor (PPI) prescribing patterns and expenditure were reviewed in different countries, including Australia, Canada, New Zealand, UK and USA.

**Results:**

Proton pump inhibitors (PPIs) are primarily indicated for control of GORD, Helicobacter pylori eradication (combined with antibiotics), preventing NSAID-induced gastrointestinal bleeding and treating peptic ulcer disease. There is widespread overprescribing of PPIs in Western and Eastern nations in terms of indication and duration, with substantial expense for national health providers. Despite a favourable short-term safety profile, there are observational associations of adverse effects with long-term PPIs. These include nutrient malabsorption, enteric infections and cardiovascular events. The prevalence of PPI use makes their long-term safety profile clinically relevant. Cost-benefit, symptom control and quality-of-life outcomes favour laparoscopic fundoplication rather than chronic PPI treatment. Laparoscopic fundoplication in long-term management of PPI-responsive GORD is supported by SAGES, NICE and ACG, and PPI-refractory GORD by AGA and SAGES guidelines. The importance of establishing a definitive diagnosis prior to invasive management is emphasized, especially in PPI-refractory heartburn.

**Conclusions:**

We examined evidence-based guidelines for PPI prescribing and deprescribing in primary care and hospital settings and the need for PPI stewardship and education of health professionals. This narrative review presents the advantages and disadvantages of surgical, endoscopic and medical management of GORD, which may assist in shared decision making and treatment choice in individual patients.

This paper was presented (GS020) at the 88th RACS Annual Scientific Conference, 6-10 May, 2019.

## Introduction

Proton-pump inhibitors (PPIs) have a favourable safety profile, are well tolerated, and are effective in treating gastro-oesophageal reflux disease (GORD), peptic ulcer disease (PUD), and non-steroidal anti-inflammatory drug (NSAID)/aspirin gastropathy [1–7]. However, there remain a number of problems and controversies in the treatment of GORD, including:

- best form of management of chronic or chronic refractory GORD;
- perceived and actual harms of long-term PPIs, as described in popular media, by national drug regulatory bodies, and in the medical literature;
- widespread inappropriate prescribing of PPIs in hospitals and primary care;
- contrasting management of GORD between gastroenterologists and surgeons;
- control of current PPI costs by governments and health service providers; and
- variations in GORD management in national and professional association guidelines.

There are differences in adverse effects of PPIs reported in large observational studies and in randomized–controlled trials (RCTs). In addition, recent developments in the surgical management of GORD, including less invasive laparoscopic or endoscopic procedures and the emerging use of robotic fundoplication, require comprehensive evaluation and comparison with PPI treatment.

This narrative literature review was based on RCTs, systematic reviews, Cochrane reports, and national/societal guidelines of the medical, endoscopic, and surgical management of GORD. It aimed to:

- review global patterns of PPI prescribing, expenditure, and potential adverse effects of chronic PPI use;
- discuss evidence-based PPI prescribing and deprescribing; and
- examine cost-effectiveness, safety profile, and outcomes of GORD treatment.

## GORD: epidemiology

The prevalence of GORD in the adult population varies by geographical location, with the highest rates reported in the USA (18.1%–27.8%), Middle East (8.7%–33.1%), Europe (8.8%–25.9%), South America (23%), and Australia (11.6%), and the lowest rates in East Asian countries (3%–8%) [5, 8, 9]. Not all of this variation can be attributed to the common risk factors for GORD (obesity, diet, alcohol consumption, tobacco smoking, aging of the population), with possible sampling, modelling, and reporting differences [5].

Risk factors for GORD can be categorized as demographic (sex, age of >65 years, obesity, race), anatomical (hiatus hernia), dysfunctional (oesophageal motility), or environmental (diet, tobacco smoking, alcohol consumption). Obesity (body mass index [BMI] > 35 kg/m^2^) and central obesity (measured by waist circumference, waist/hip ratio, visceral fat area) are independent risk factors for erosive oesophagitis. Risk factors for Barrett’s oesophagus include chronic GORD, hiatus hernia, male sex, age of >50 years, Caucasian race, tobacco smoking, central obesity, and family history of Barrett’s oesophagus or oesophageal adenocarcinoma. Males are much more likely to have Barrett’s oesophagus than females (M:F = 2–4:1) and develop oesophageal adenocarcinoma (M:F = 8:1). Between 1995 and 2006, there was a 7-fold increase in the US incidence of oesophageal adenocarcinoma, driven by GORD and central obesity [4, 9–11].

## GORD: pathophysiology and presentation

GORD is associated with disorders of the oesophagogastric junction (OGJ) and failure to clear oesophageal refluxate or neutralize it by salivary bicarbonate. The anti-reflux barrier is a combination of lower oesophageal sphincter (LOS), diaphragmatic crural sling, phreno-oesophageal ligament, angle of His, and the length of the intra-abdominal oesophagus [1, 6]. Disruption of the anti-reflux barrier increases the risk of GORD. This can occur due to transient LOS relaxation (TLOSR), hiatus hernia, or hypotensive LOS.

Central obesity leads to raised intragastric pressure, a mechanically defective LOS, and increased TLOSR events. Postprandial reflux can occur in both healthy and GORD patients due to more frequent TLOSR, related to meal-induced gastric accommodation and gastric acid secretion. Ineffective oesophageal motility (IOM) is associated with impaired oesophageal acid and bolus clearance, and is found in achalasia, scleroderma, oesophageal hypocontractility, and acid reflux. However, impaired acid clearance is only seen in severe IOM (>80% abnormal peristaltic sequences) and is not associated with mild IOM in patients with GORD. There is a higher prevalence of hiatus hernia, IOM, and abnormally low LOS pressure in patients with GORD compared with healthy control patients and those with functional heartburn [6].

The commonly described symptoms of GORD include heartburn and regurgitation. Atypical or extra-oesophageal symptoms may include non-cardiac chest pain, halitosis, sore throat, globus pharyngeus, nocturnal cough, sleep disturbance, wheeze, hoarseness, throat clearing, or nausea. Extra-oesophageal manifestations of GORD may include laryngitis, pharyngitis, dysphonia, asthma, chronic bronchitis, and aspiration pneumonia [1, 10, 12, 13]. Oesophageal complications of GORD include distal oesophageal stricture, ulceration, and haemorrhage. These can potentially be prevented by PPI treatment. Whether progression of Barrett’s oesophagus to high-grade dysplasia or oesophageal adenocarcinoma is preventable by PPI therapy or anti-reflux surgery is debated [1, 10, 12].

Among patients with typical heartburn symptoms, up to 30% have endoscopic findings of erosive reflux disease or Barrett’s oesophagus [13]. Thus, the majority of patients who present with typical reflux symptoms have non-erosive reflux disease (NERD) on endoscopy. Distinguishing true NERD from functional heartburn or reflux hypersensitivity is important, as they share a macroscopically normal oesophageal appearance at endoscopy but require different management. Differentiation of these heartburn phenotypes is defined in the Rome IV classification [14]. Reflux hypersensitivity is related to increased perception of chemical (refluxate, acid), mechanical (oesophageal distension), electrical, or thermal stimuli. This is mediated by both peripheral and central sensitization, worsened by psychological stress or sleep deprivation, and characterized by oesophageal hypervigilance and visceral hyperalgesia [6, 14]. Acid reflux and bile acids work in synergy to produce oesophageal mucosal inflammation, with acidic bile acids being involved in progression of Barrett’s oesophagus. Although the presence of dilated intercellular spaces in squamous epithelium on endoscopic biopsy/histopathology is not specific for NERD, it may indicate increased oesophageal mucosal permeability and exposure of submucosal nociceptive chemoreceptors to acidic or bile acid reflux [6, 14, 15].

## GORD: PPI prescribing

When comparisons are made between PPI therapy and invasive interventions for GORD, it is important to consider not only treatment efficacy but also cost-effectiveness and adverse events. Annual direct healthcare costs for PPIs exceed US$10 billion in the USA and £425 million in the UK [5, 16]. In Australia, from 30 June 2020 to 1 July 2021, there were 15,327,811 Pharmaceutical Benefits Scheme (PBS)-subsidized prescriptions filled for pantoprazole, esomeprazole, rabeprazole, or omeprazole, costing $252,274,196 (government + patient contribution in Australian dollars) [2]. Use of PPIs increased by 1,318% in Australia between 1995 and 2006 [4]. The rate of increase in PPI use slowed to 5% between 2007 and 2017 [2, 17]. Similar patterns of increased prevalence but relatively stable incidence of PPI use occurred in the USA, Europe, and the UK during this time period, with the majority of outpatient prescriptions for PPIs being chronic repeats rather than new prescriptions [17, 18]. In Australia in 2001, PBS restrictions were relaxed, which enabled PPI prescribing to occur for lesser symptoms or non-indicated reasons [4]. In 2015, approval was given to Australian pharmacies to dispense over-the-counter low-dose pantoprazole without a prescription. This was expanded to standard strength esomeprazole and low-dose rabeprazole and lansoprazole in 2016 [2].

### PPI prescribing patterns

In 2015, GORD represented 13.2% of all new general medical practice presentations in Australia and ranked 11th amongst managed conditions [3]. Prescribing of PPIs in elderly patients (>75 years) is substantially more prevalent than in younger patients, in part related to aspirin-based antiplatelet or NSAID therapy and PUD/upper gastrointestinal bleeding prophylaxis [7]. Older patients may be more susceptible to the potential harms of long-term PPI use, including polypharmacy, adverse effects, and cost [2, 4, 16, 19]. Inappropriate PPI prescribing is prevalent in many Western and Eastern nations [16, 18–20]. In Australia, Greece, Spain, the UK, and the USA, up to 81% of hospital inpatients and 55% of primary care patients who are prescribed PPIs have no proper indication for their use [16, 18].

It is estimated at least £100 million annually is spent by the UK National Health Service on unnecessary PPI use [16]. In one study of hospital inpatients taking PPIs in Australia, 63% did not meet the PBS criteria, while among inpatients in New Zealand, 40% were prescribed a PPI inappropriately, two-thirds of whom were later discharged on PPIs. Most were still taking PPIs 6 months later [16]. In another study conducted in a tertiary hospital in Malaysia, empirical intravenous PPIs were most commonly prescribed for unexplained abdominal pain in 76.4% of patients, mainly by junior doctors on the medical (39.6%) and surgical wards (44.3%) [21]. Among patients who received intravenous PPIs, 53% were found to be inappropriately prescribed, based on duration (35.8%), dose (32.1%), or indication (32.1%) [21]. In Singapore, of 1,025 patients admitted to hospital on a randomly selected day, 46.5% were prescribed PPIs. In 54% of these prescriptions, the US Food and Drug Administration (FDA) PPI indications were not fulfilled [18]. Specified indications for PPI commencement, recommended treatment duration, and a suggested review date are frequently lacking in hospital discharge summaries [16]. This prescribing documentation requires improvement via health professional education and better links to primary care physicians [19].

### Australian PBS intervention

The Australian PBS responded to overprescribing of high-dose PPIs for excessively long periods by reintroducing prescribing restrictions in May 2019 [17]. All high-dose PPI formulations, and both high- and low-dose esomeprazole, became “Authority Required” with limited repeats. Subsidized PPIs include omeprazole, lansoprazole, rabeprazole, pantoprazole, and esomeprazole. Main subsidized indications were GORD, PUD, and *Helicobacter pylori* eradication. Other indications included scleroderma oesophagus, Zollinger–Ellison syndrome, and idiopathic hypersecretory disorders [2, 17]. GORD pathology had to be inadequately controlled by a low-dose PPI for a higher dose to be prescribed. The 2019 PBS intervention has resulted in a decrease in new prescriptions for high-dose PPIs, but not total number of PPI prescriptions or PBS-subsidized expenditure, according to comparisons of 2018–19, 2019–20, and 2020–21 fiscal years [2].

## PPI adverse effects

PPIs are generally well tolerated and have a favourable safety profile with short-term use. Common adverse effects include abdominal pain, diarrhoea, nausea, dizziness, and headaches (<2%), but these are infrequently the reason for PPI cessation. Guidelines suggest that after effective GORD treatment, the majority of patients do not require long-term PPI therapy, except for select indications such as severe erosive oesophagitis (Los Angeles [LA] Grade C or D) or Barrett’s oesophagus [19]. Epidemiological and experimental studies report multiple adverse effects of chronic PPI use, which differ from findings of existing RCTs [22]. Risk of adverse events is an important consideration, particularly in patients chronically taking PPIs for unspecified indications (undifferentiated abdominal pain, non-ulcer dyspepsia, functional heartburn) with limited benefit. However, this should not change PPI prescribing for evidence-based indications [1, 23–25].

Canada Health issued health warnings about PPI-related adverse drug reactions involving severe hypomagnesaemia, hypokalaemia, and hypocalcaemia (in 2011), *Clostridioides difficile* colitis (in 2012), bone fractures (in 2013), and drug interactions with clopidogrel (in 2009) and methotrexate (in 2012). The US FDA released similar warnings about PPIs and increased risks of *C. difficile* infection, bone fractures, and severe hypomagnesaemia. In 2009, the US FDA issued warnings about concomitant prescribing of clopidogrel with omeprazole/esomeprazole due to concerns about impaired inhibition of platelet aggregation. The warning did not extend to other PPIs [26].

However, the Clopidogrel and the Optimization of Gastrointestinal Events Trial (COGENT) published in 2010 showed a significant reduction in gastrointestinal bleeding without any increased cardiovascular events after 180 days in patients with acute coronary syndrome (ACS) treated with clopidogrel/aspirin (dual antiplatelet therapy [DAPT]) and randomized to receive 20 mg daily of omeprazole. There are differences in subsequent expert guidelines due to interpretation of the COGENT findings. For example, the 2016 American College of Cardiology/American Heart Association guidelines recommend DAPT with low-dose aspirin and a P2Y12 inhibitor (clopidogrel, ticagrelor, or prasugrel) after ACS or percutaneous coronary artery intervention (PCI) and drug eluting stent be co-prescribed with PPI prophylaxis only in patients at high risk of gastrointestinal bleeding. The European Society of Cardiology 2018 guidelines recommend routine co-prescription of PPIs for all patients receiving DAPT. The European Society of Cardiology guidelines also commented that although omeprazole did not appear to increase the risk of adverse cardiovascular events, pantoprazole and rabeprazole had the lowest propensity for clinically relevant drug–drug interactions as compared with omeprazole and esomeprazole [7, 24, 25].

### PPI-induced hypochlorhydria and hypergastrinaemia

**Figure 1.**
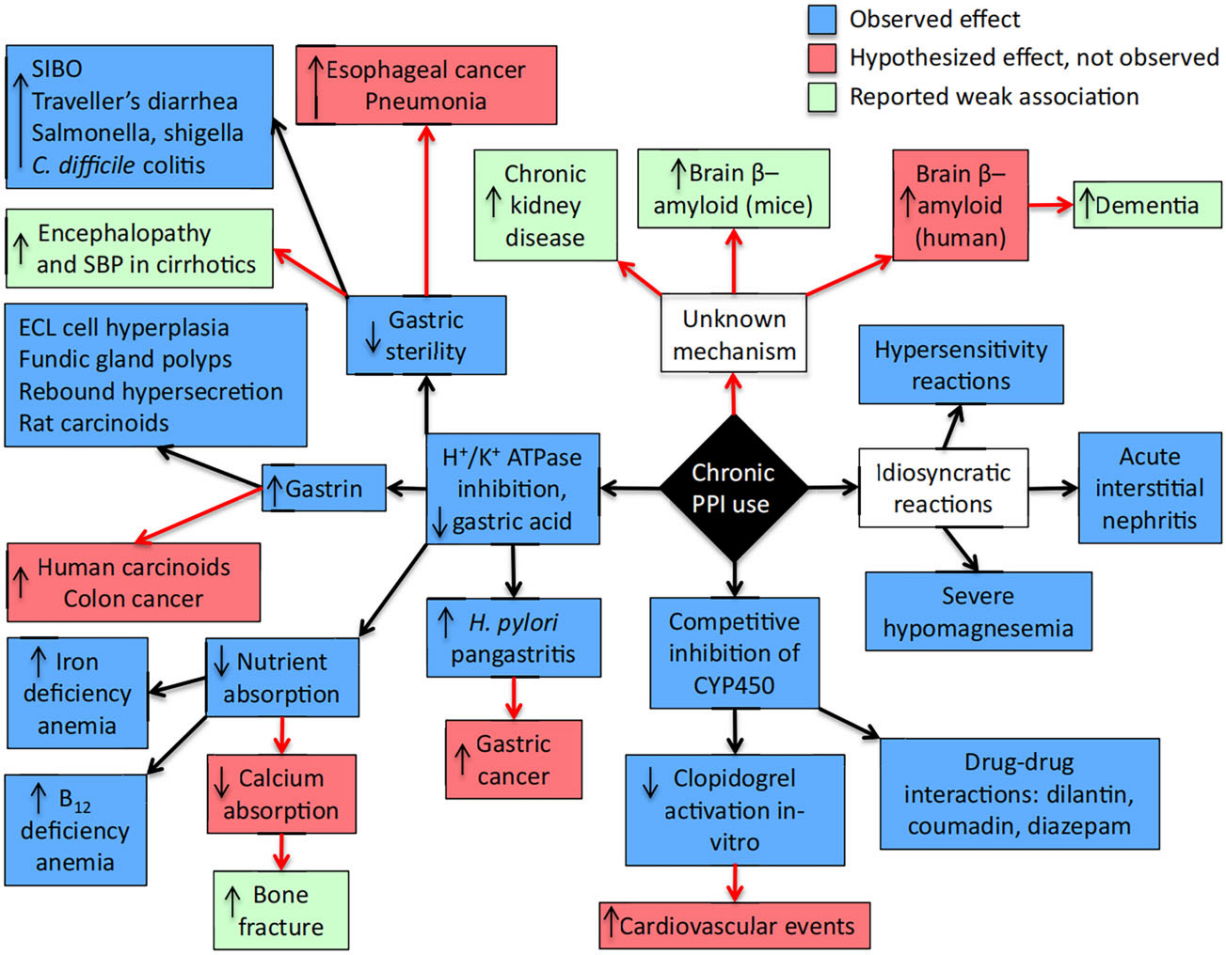
Mechanisms of potential risks associated with PPIs [24]. Blue boxes represent effects that are observed. Green boxes represent effects that are weakly associated with PPI use. Pink boxes represent hypothetical effects that have not been recognized or observed in association with PPI use. Black arrows are established linkages, whereas red arrows are proposed unproved links. ECL, enterochromaffin-like; SBP, spontaneous bacterial peritonitis; SIBO, small intestinal bacterial overgrowth.

Population prevalence of PPI use makes the long-term safety profile of PPIs clinically relevant, even if the attributable risk is modest. This is known as population-attributable risk [1, 25]. Some of the potential risks have observed effects with plausible mechanisms (Figure 1) [23–25, 27]. PPI-induced hypochlorhydria inhibits the release of somatostatin from gastric antral D-cells. Somatostatin has an inhibitory effect on gastrin release from antral G-cells and histamine release from enterochromaffin-like cells. Thus, chronic PPI therapy results in a 2- to 6-fold increase in serum gastrin levels in 80%−100% of patients. Gastrin normally stimulates the release of gastric hydrochloric acid via activation of cholecystokinin type B/gastrin receptors on gastric parietal cells. Gastrin also stimulates the release of histamine from gastric enterochromaffin-like cells, which indirectly acts as a paracrine stimulus of gastric hydrochloric acid secretion via activation of parietal cell histamine type-2 receptors. Gastrin stimulates mitosis, RNA, DNA, and cell membrane protein synthesis, and is a trophic factor for both parietal and enterochromaffin-like cells. PPI-induced enterochromaffin-like cell hyperplasia results in elevated circulating histamine and chromogranin A levels (which usually resolve after PPI cessation) in humans and gastric carcinoid tumours in rats. The role of chronic hypergastrinaemia in the development of Barrett’s oesophageal adenocarcinoma is controversial [18, 23, 27].

### PPI adverse effects: nutrient absorption and gut flora

Decreased gastric acid production also affects nutrient absorption and gut flora, potentially leading to iron deficiency anaemia (odds ratio [OR]: 2.49), hypomagnesaemia (relative risk [RR]: 1.44), calcium, zinc, vitamin C, and B12 malabsorption (hazard ratio [HR]: 1.83), thiamine antagonism, small intestinal bacterial overgrowth (OR: 1.71), and enteric infections (OR: 4.28) [22, 23, 28–40]. Up to 13% of patients on PPIs long-term (mean 5.5 years) develop hypomagnesaemia (Mg < 0.7 mmol/L). PPIs interfere with claudin-dependent *paracellular* magnesium absorption in the small intestine. In addition, PPIs impair *active* magnesium transport in the distal ileum and colon by inhibiting transient receptor potential cation channel subfamily melastatin member 6 (TRPM6), an epithelial magnesium transporter. Research has also shown that patients with single nucleotide polymorphisms in TRPM6 expression are 5.8 times more likely to develop PPI-induced hypomagnesaemia [30, 34, 39, 40].

**Figure 2.**
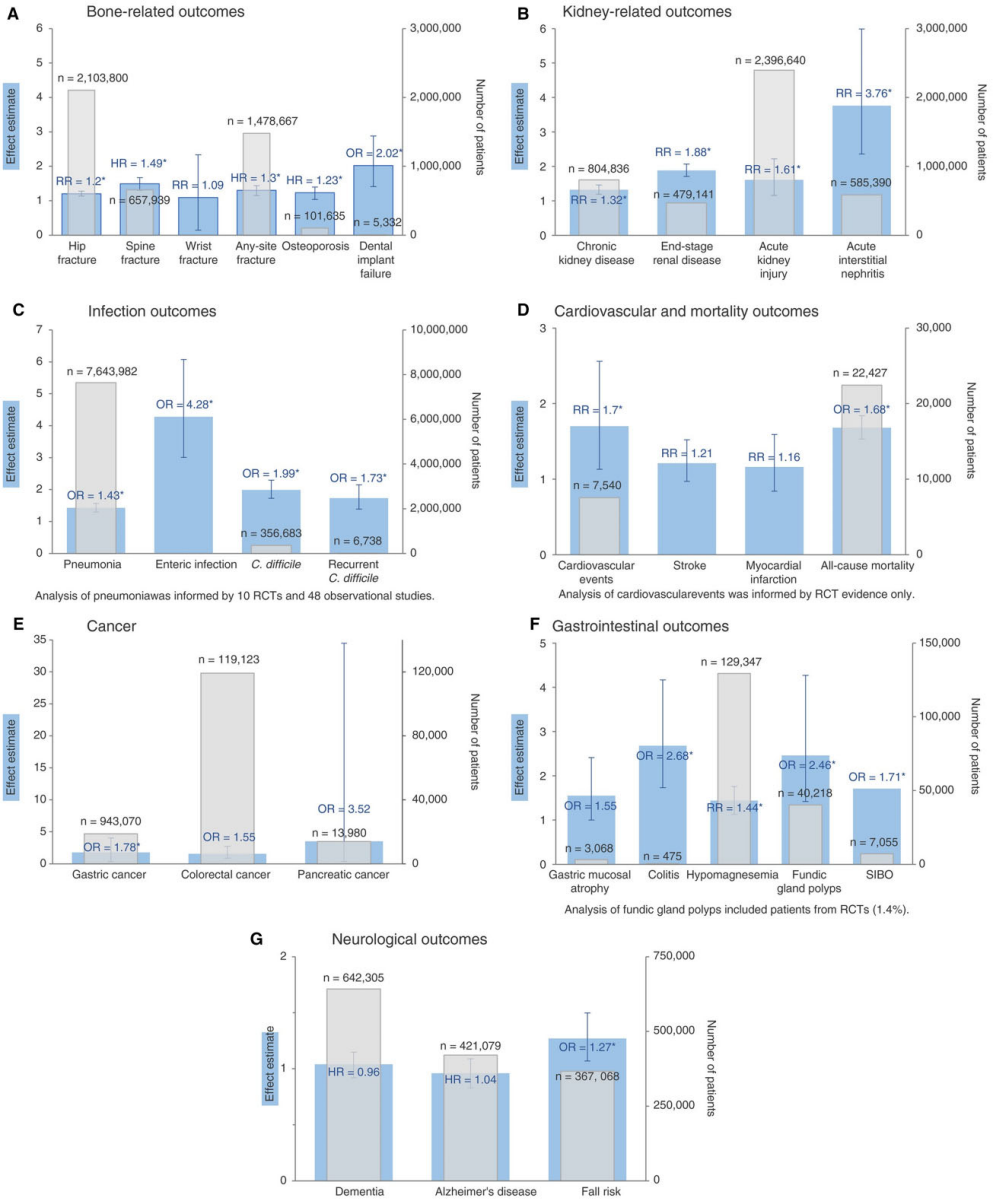
Summary of evidence for the associations between PPI use and adverse outcomes in systematic reviews with meta-analyses categorized as the most comprehensive for each outcome [31]. Bar charts are presented based on outcome type: (A) bone-related outcomes, (B) kidney-related outcomes, (C) infection outcomes, (D) cardiovascular and mortality outcomes, (E) cancer, (F) gastrointestinal outcomes, (G) neurological outcomes. Data are based on observational studies, unless otherwise noted. *Associations found to be statistically significant for PPI use and adverse outcome (*P *<* *0.05).

Enteric pathogens, including *Salmonella* spp., *Campylobacter jejuni*, enteropathogenic *Escherichia coli*, *Shigella* spp., and the vegetative forms of *C. difficile*, are usually unable to survive the acidic gastric environment (pH = 1). However, PPIs raise intragastric pH to >4, and inhibit neutrophil and colonic epithelial tight junction barrier function. This enables enteric pathogens to survive and cause enteritis or colitis. PPIs also raise the pH of the duodenum and jejunum, which enhances *C. difficile* spore germination and vegetation in the presence of primary bile salts. The prevalent use of PPIs is associated with both hospital-acquired and community-acquired *C. difficile* colitis [31, 32, 35] (Figure 2).

PPIs can alter the healthy resident microbiota of the gastrointestinal tract, leading to small intestinal bacterial overgrowth and intestinal colonization by multidrug-resistant organisms (vancomycin-resistant *Enterococcus*, carbapenemase-producing *Enterobacter*, extended-spectrum *β*-lactamase-producing *Enterobacter*) [37]. Hypochlorhydria can also result in gastric dysbiosis, growth of nitrate reductase producing bacteria and endogenous bacterial *N*-nitrosation at pH 5–8 [38].

Gastric acidity is required for the dissociation and subsequent absorption of inorganic salts (MgO, CaCO_3_) and organic acids (acetyl salicylic acid, ascorbic acid), and extraction of ligand-bound nutrients from food (vitamin B12, zinc, non-heme iron) [7, 30, 31, 33, 38].

In 2010, the US FDA issued a safety warning about increased risk of spine, wrist, and hip fractures with PPI use, although a direct causal relationship has not been proven [29]. Human observational and experimental animal studies have reported lower trabecular bone score, poor bone mineralization and osteoporosis (HR: 1.23), bone fractures (HR: 1.3), and dental implant failure (OR: 2.02) associated with PPI treatment [31]. Suggested mechanisms related to PPIs include:

- dysregulation of the balance between bone osteoclastic and osteoblastic activity;
- hypergastrinaemia-induced parathyroid gland hyperplasia;
- hypergastrinaemia-induced histamine secretion causing osteoclastogenesis and bone resorption;
- impaired bone collagen production [29];
- inhibition of the vacuolar type of H+-ATPase of bone osteoclasts leading to bone resorption and increased release of deoxypyridinoline [36];
- hypochlorhydria-induced calcium malabsorption and secondary hyperparathyroidism;
- inadequate hydroxylation of vitamin D intermediates by 25-hydroxycholecalciferol-1-hydroxylase due to PPI-induced hypomagnesaemia; and
- decreased osteoblastic activity due to hypomagnesaemia [23, 24, 28–31, 36].

PPIs are only weakly associated with such side effects as bacterial translocation, spontaneous bacterial peritonitis, and hepatic encephalopathy in cirrhotic patients. Acute interstitial nephritis in association with PPI use (RR: 3.76) has been described as an idiosyncratic reaction, which may develop into chronic kidney disease (CKD) if unrecognized or left untreated due to chronic renal interstitial scarring and tubular atrophy [1, 23, 31]. There are only weak associations between PPI use and development of Alzheimer’s disease (HR: 1.04) or CKD (RR: 1.32) (Figures 1 and 2) [22, 24, 25, 31].

### Cytochrome P450 (CYP)2C19 and relationship with PPI adverse effects

All PPIs, except for rabeprazole, are primarily metabolized by CYP2C19 and, to a lesser extent, CYP3A4, which are hepatic cytochrome P450 isoenzymes. Esomeprazole is a second-generation PPI that is less affected by CYP2C19 (70% metabolized) than the first-generation PPIs omeprazole, pantoprazole, and lansoprazole (80%–90% metabolized). Omeprazole and its S-enantiomer esomeprazole both share non-linear pharmacokinetics related to auto-inhibition of CYP2C19, which increases their bioavailability and gastric acid suppression after repeated doses. A side effect of this inhibition of CYP2C19 is decreased formation of the active metabolite of clopidogrel, which is clopidogrel thiol H4 (Clo-AM). Clo-AM irreversibly binds to the platelet P2Y12 receptor resulting in inhibition of adenosine diphosphate (ADP)-induced platelet aggregation. Attenuation of this antiplatelet effect of clopidogrel by omeprazole and potential cardiovascular thrombotic events was the basis for the US FDA 2009 safety warning [40, 41]. Complicating this proposed interaction between PPIs and clopidogrel are genetic polymorphisms in CYP2C19. Across ancestral groups, loss-of-function genetic polymorphisms in CYP2C19 occur in 5%–15% of Caucasian and African (CYP2C19*2), 23%–35% of Asian, and 35%–70% of Pacific Islander (CYP2C19*2,*3) people, characterized by a poor metabolizer phenotype. This results in greater PPI bioavailability, prolonged gastric acid suppression, decreased clopidogrel activation, and potentially more frequent side effects in certain patient populations, including enteric infections and cardiovascular events (ischaemic stroke, acute myocardial infarction [AMI]). Conversely, 18%–28% of Caucasian, 17%–18% of African, and 0.3%–4% of Asian people have gain-of-function mutations in CYP2C19 (CYP2C19*17) that increase the enzyme activity. This results in an ultra-rapid metabolizer phenotype and potential *decreased efficacy* of pantoprazole, omeprazole, or lansoprazole in treating erosive oesophagitis or gastroduodenal ulceration. Thus, polymorphisms in CYP2C19 may explain some of the variability in GORD symptom control and potential adverse side effects across differing PPI drugs and patient populations [22, 32, 39, 41].

### PPIs and cardiovascular adverse events

Associations between PPI use and increased mortality, cardiovascular events, carcinoid tumours, and gastric, oesophageal, pancreatic, colon, and primary liver cancer have been reported in some experimental studies and systematic reviews, although causation has not been established [22, 26, 27]. A 2021 systematic review and meta-analysis included six RCTs involving 4,675 patients and 22 non-randomized studies involving 126,708 patients treated with clopidogrel after PCI. From the meta-analysis of the 28 pooled studies, the concomitant use of PPIs was associated with increased risk of major adverse cardiovascular end points (MACE) (RR: 1.30, 95% confidence interval [CI], 1.15–1.48; *P *<* *0.001, *I*^**2**^* *=* *89.0%) and AMI (RR: 1.43, 95% CI, 1.25–1.64; *P *<* *0.001, *I*^**2**^* *=* *61%). When only the six RCTs were pooled, there was no increased MACE risk with PPIs and no heterogeneity between studies (RR: 1.01; 95% CI, 0.82–1.24; *I*^**2**^* *=* *0). MACE were a composite of cardiovascular death, AMI, stroke, and revascularization. There was an overall increased risk of MACE for all four PPIs that competitively inhibit CYP2C19 activity (esomeprazole, omeprazole, lansoprazole, and pantoprazole), but not for rabeprazole [42]. Reduction in upper gastrointestinal bleeding by the addition of PPIs to DAPT after PCI or ACS (RR: 0.40, 95% CI, 0.24–0.64, *P *<* *0.001, *I*^**2**^* *=* *0%) still outweighed the risks of PPI-related MACE. Low-dose PPIs may be associated with lower MACE compared with high-dose PPIs, but this remains to be proven [39, 42].

Pathways other than competitive inhibition of clopidogrel activation have been proposed to explain the increased incidence of MACE [40], including PPI-induced:

- impairment of endothelial nitric oxide synthase and nitric oxide (NO) production, due to PPI inhibition of dimethylarginine dimethylaminohydrolase and accumulation of asymmetrical dimethylarginine;
- impairment of (a) formation of gastric NO from nitrous acid and inorganic nitrate, and (b) formation of gastric S-nitrosothiols (a NO donor);
- interference with vitamin C absorption resulting in activation of reactive oxygen species-dependent pathways, which results in oxidation of endothelial tetrahydrobiopterin, uncoupling of endothelial nitric oxide synthase, less NO formation, and greater superoxide and peroxynitrite production;
- vitamin B12 deficiency and elevated homocysteine and asymmetrical dimethylarginine levels;
- inhibition of endothelial lysosomal proton pumps, leading to oxidative stress, telomere shortening and accelerated endothelial senescence, and endothelial-to-mesenchymal transition [39]; and
- elevation of circulating chromogranin A levels and endothelin-1 release, and accelerated atherosclerosis and cardiovascular disease [39, 40].

However, the Cardiovascular Outcomes for People Using Anticoagulation Strategies (COMPASS) study found a significantly increased risk of enteric infections only (OR: 1.33, 95% CI, 1.01–1.75; *P *=* *0.04) with PPI use [26]. This was a large, multi-centre, double-blind, randomized, placebo-controlled trial of 40 mg/day of pantoprazole combined with aspirin/rivaroxaban in 17,598 patients with stable cardiovascular disease who were followed for a median of 3 years. There was no increased risk of cardiovascular events, pneumonia, fractures, dementia, CKD, cancer, or all-cause mortality in participants randomized to receive pantoprazole vs placebo. Criticisms of the COMPASS study include the comparatively short PPI (pantoprazole) exposure time, short follow-up and inadequate power to detect some low event outcomes, including mortality, cancer, and CKD [23, 26, 44]. Only referring to evidence from RCTs also contravenes the principles of “real-world” pharmacovigilance of serious adverse drug reactions [26]. Differences in PPI adverse risks between meta-analyses may be related to inclusion criteria, residual confounding risk factors, and date of publication [31].

The American College of Gastroenterology (ACG) 2022 GORD management guidelines warned of the difference between “association” and “causation” in relation to PPI adverse effects [1]. Based on 1965 Bradford Hill epidemiological criteria, cause-and-effect conclusions should not be drawn in observational studies unless the RR in cohort studies exceeded 2–3 or the OR in case–control studies exceeded 3–4. This is because of confounding lifestyle factors, *channeling* bias or *protopathic* bias in observational studies compared with well-conducted RCTs. PPI use can be a *surrogate* marker for pre-existing patient comorbidities, such as obesity, hyperlipidaemia, diabetes, advanced age, *Helicobacter pylori* gastritis, CKD, chronic obstructive lung disease, or ischaemic heart disease, rather than a truly causative factor in adverse outcomes [1, 24, 39].

### PPIs and gastric cancer

There is a stronger association between PPI use and gastric cancer than with non-gastric cancers. This is confounded by *H. pylori* infection and CagA virulence, alcohol consumption, tobacco smoking, diet, age, sex, race, and family history in observational studies of PPI-related gastric carcinogenesis [22, 23, 31, 43, 44]. *Helicobacter pylori* infection is involved in both the Correa pathway of intestinal-type gastric adenocarcinoma and the pathogenesis of sporadic diffuse-type gastric adenocarcinoma [38, 43, 44]. PPIs appear to worsen chronic atrophic corpus gastritis in patients with *H. pylori*, but not *H. pylori*-negative patients [23, 43]. A 2019 meta-analysis of 926,386 pooled participants from East Asian and Western countries identified a 2-fold increased risk of gastric cancer with long-term PPI use after *H. pylori* eradication (OR: 2.1; 95% CI, 1.1–3.09) [43]. The risk was higher in the subgroup analysis of 66,052 Asian patients (OR: 2.44, 95% CI, 1.89–3.00) as compared with the 860,334 Caucasian patient subgroup (OR: 1.86; 95% CI, 0.54–3.18). The duration–response relationship revealed that the risk of gastric cancer was increased if PPI use lasted for >1 year [43].

A significant association with gastric cancer and PPI use was also found in a Hong Kong population-based study of 63,397 patients after *H. pylori* eradication treatment, with a median follow-up of 7.6 years [44]. This was more evident in non-cardia gastric adenocarcinoma (OR: 2.45, 95% CI, 1.44–3.45) than in cardia gastric adenocarcinoma (OR: 1.64, 95% CI, 0.23–3.51). Risk of gastric cancer progressively increased with PPI frequency of use: at least weekly (HR: 2.43, 95% CI, 1.37–4.31) or daily (HR: 4.55, 95% CI, 1.12–18.52) compared with less than once per week [43, 44]. Gastric cancer risk also increased with PPI duration of use: 1 year (HR: 5.04, 95% CI, 1.23–20.61), 2 years (HR: 6.65, 95% CI, 1.62–27.26), 3 years (HR: 8.34, 95% CI, 2.02–34.41). Use of histamine-2 receptor antagonists (H2RAs) did not exhibit the same association. Proposed mechanisms relate to chronic hypochlorhydria induced by PPIs, resulting in gastric dysbiosis, increased endogenous *N*-nitrosamine formation, hypersecretion of gastrin, activation of JAK–STAT signalling, and enterochromaffin-like cell hyperplasia (Figure 2) [23, 27, 38, 43, 44]. Intestinal-type gastric cancer progression is more likely in patients with previously treated *H. pylori* and persistent chronic atrophic gastritis or intestinal metaplasia, but the Hong Kong study did not include detailed baseline gastric histology [44]. It has also been suggested that signet ring cell (diffuse-type) gastric adenocarcinoma may develop in de-differentiated neuroendocrine tumours, due to prolonged hypochlorhydria, hypergastrinaemia, and enterochromaffin-like hyperplasia associated with autoimmune pernicious anaemia, prolonged PPI therapy, or chronic atrophic gastritis [23, 27, 44].

Similarly, a 2021 UK population-based study reported that PPI use was associated with a 45% increased risk of gastric cancer compared with the use of H2RAs (HR: 1.45, 95% CI, 1.06–1.98) over a median time of 5 years. HRs increased with cumulative omeprazole equivalents, cumulative duration of use, and time since initiation of treatment. However, absolute risk remained low [45]. A 2021 meta-analysis of 13 observational studies involving 1,662,881 individuals also reported an association between PPIs and development of non-cardia gastric cancer (OR: 2.20, 95% CI, 1.44–3.36, *I*^**2**^* *=* *77%), but could not confirm causation due to study heterogeneity. It recommended that clinicians carefully evaluate PPI prescribing practices, particularly in high-risk patients, and only prescribe PPIs when strictly indicated [46].

## GORD medical management

Short-term PPIs are highly effective in the treatment of GORD. PPIs have superior gastric acid suppression, erosive oesophagitis healing rates (12%/week vs 6%/week), and control of GORD symptoms (11.5%/week vs 6.4%/week) as compared with histamine-2 receptor antagonists (H2RAs) [1]. In long-term maintenance treatment of complicated GORD, PPIs were found to be superior to H2RAs in the prevention of recurrent oesophagitis (80% vs 49%) and oesophageal stricture (46% vs 30%) [7]. Patients who respond well to PPI therapy exhibit improvements in GORD health-related quality of life (HRQL), but this does not occur in PPI non-responders [10]. Large-volume regurgitation, atypical symptoms, and functional disorders usually respond poorly to PPI treatment. A high-dose PPI trial for 4–8 weeks is recommended for GORD and 8–12 weeks for PUD [1, 14, 47]. Long-term high-dose PPI use has limited and restricted indications [6, 10, 47].

### Lifestyle changes

Modification of lifestyle risk factors should complement gastric acid suppression (PPI therapy) as first-line management of GORD. Patients should be counselled regarding obesity and weight loss, meal size, and frequency, avoiding postprandial recumbent positions, reducing alcohol intake, cessation of smoking, and avoiding specific foods (fatty/acidic foods, caffeine/chocolate) or beverages (tea, coffee, carbonated drinks) that exacerbate symptoms [1, 10]. A BMI decrease of ≥3.5 kg/m^2^ is associated with a 40% decrease in GORD symptoms in women [1]. A diet including psyllium fibre and plant-based protein instead of animal-based protein may be beneficial in improving LOS resting pressure and decreasing acid reflux and postprandial symptoms [6]. Review of medications that promote GORD (anticholinergics, benzodiazepines, beta-2 adrenergic agonists, oral bisphosphonates, calcium channel blockers, nitrates, NSAIDs, opioids) is useful [6, 48].

### Optimizing PPI therapy

Poor patient compliance, inadequate PPI dose, and incorrect timing of PPI dose can all contribute to refractory reflux symptoms, with up to 54% of patients incorrectly taking their PPIs. To achieve their maximal effect, esomeprazole and lansoprazole should be taken on an empty stomach, as food decreases their bioavailability. All PPIs are prodrugs and require protonation to become activated. This occurs in the gastric parietal cell secretory canaliculi, where PPIs then accumulate at concentrations 1,000 times greater than plasma. PPIs are very effective (95%), but only when proton pumps are actively secreting acid. PPIs have a short plasma half-life (90 min) but a long biological activity and should ideally be taken 30–60 min before meals. This is so maximal plasma concentrations are achieved at the same time as proton pumps are activated by food [1, 6, 47].

This biological longevity is due to activated PPIs covalently and irreversibly binding to proton pumps. Restoration of gastric acid secretion requires de novo synthesis of hydrogen/potassium adenosine triphosphatase (H^+^/K^+^-ATPase) proteins. Greater synthesis of new H^+^/K^+^-ATPase proteins occurs during overnight fasting than during the daytime, which is another rationale for morning PPI dosing. Only 70% of proton pumps are activated by a breakfast meal, such that steady-state inhibition with once-daily pre-breakfast PPI achieves a 66% reduction in maximal gastric acid output after 5 days. However, “split” dosing of PPIs before breakfast and dinner can decrease maximal gastric acid output by 80%, which may be useful for nocturnal reflux symptoms or more severe GORD [1, 6, 47].

Using the standardized effect of omeprazole on 24-hour gastric acid production, the comparative potencies of PPIs are, respectively, 0.23, 0.90, 1.00, 1.60, and 1.82 for pantoprazole, lansoprazole, omeprazole, esomeprazole, and rabeprazole [1, 30]. Switching to a more potent, CYP-independent PPI (e.g. rabeprazole) may result in improved efficacy in some patients. Adding an H2RA at night to a twice-daily PPI dosage can improve nocturnal acid breakthrough from 64% to 17%, as histamine drives nocturnal acid production. This, however, is associated with rapid H2RA tachyphylaxis. Intermittent or on-demand H2RA use may be preferable. Postprandial alginates can be useful as an adjunct to PPIs for controlling residual GORD symptoms. The addition of prokinetic agents (domperidone, metoclopramide, mosapride) is not recommended unless there is objective evidence of gastroparesis. This is due to lack of efficacy of prokinetic agents in GORD treatment and risk of side effects including QTc prolongation or dystonia [1, 6, 47].

### PPI deprescribing

In the setting of a positive clinical response to a trial of high-dose PPIs, deprescribing guidelines recommend weaning to the lowest effective dose or an on-demand basis [5, 19]. Oral antacids, alginates, or H2RAs may be helpful during this taper period [19]. Weaning off PPIs prevents rebound gastric acid hypersecretion observed with abrupt cessation, related to PPI-induced hypergastrinaemia [10]. Deprescribing is not appropriate in patients with historical bleeding PUD, Barrett’s oesophagus, peptic stricture, or severe erosive oesophagitis (LA Grade C or D) [19]. A Swedish double-blind, placebo-controlled RCT of PPI deprescribing found that 27% of patients taking PPIs for 4 years were able to completely cease PPIs [16]. Hospital PPI stewardship by senior clinicians and hospital pharmacists can help prevent inappropriate empirical initiation of PPIs by junior medical staff when patients are hospitalized and perpetuation in the community when patients are discharged [20, 21].

### Assessment of persistent symptomatology

**Figure 3.**
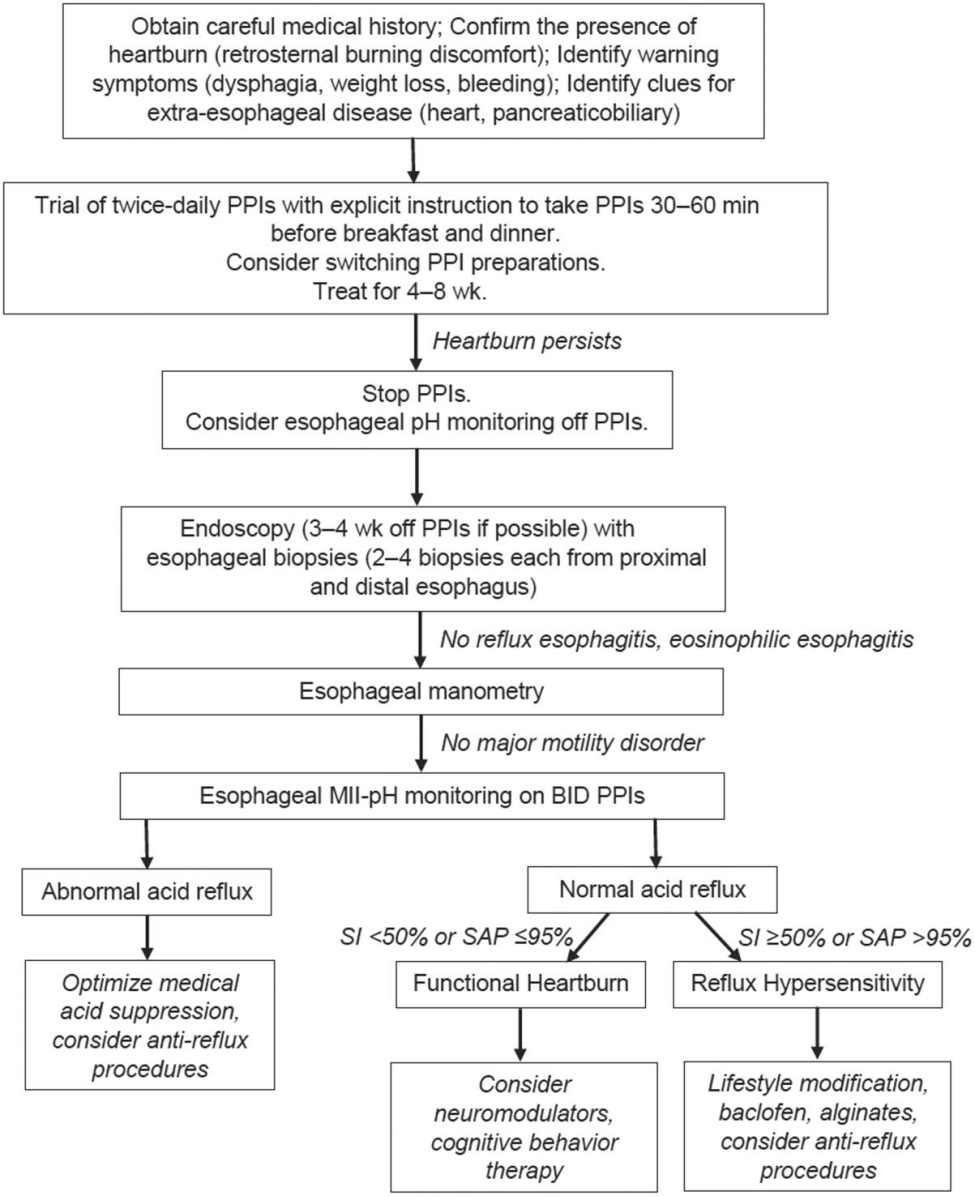
Approach to the management of patients with heartburn refractory to PPI therapy [47]

Patients with persistent symptoms despite a trial of PPI therapy should be investigated with endoscopy/mucosal biopsy and oesophageal pH manometry to exclude GORD complications or alternate pathology. Such a workup involves assessment for *H. pylori*, oesophagogastric malignancy, PUD, oesophageal stricture, hiatus hernia, eosinophilic oesophagitis, and digestive, functional, or motility disorders [6, 10, 15]. Endoscopy should also be performed in patients with GORD symptoms and multiple risk factors for Barrett’s oesophagus. Cessation of PPIs for at least 2 weeks prior to endoscopy may assist in the diagnosis of erosive oesophagitis or eosinophilic oesophagitis. This is important in the diagnosis of NERD, as up to 10% of patients with NERD may have healed erosive oesophagitis [1, 47, 48]. Alarm symptoms, including dysphagia, odynophagia, haematemesis, iron deficiency, or involuntary weight loss should be promptly and extensively investigated with endoscopy and imaging [10, 47]. Assessment of extra-oesophageal symptoms should include otorhinolaryngology, respiratory, or immunology/allergy specialists due to overlap with other pathological conditions and variable responses to GORD treatments in these patients. Functional disorders are characterized by absence of both erosive oesophagitis and pathological acid exposure, and may be improved by pharmacological neuromodulation (low-dose selective serotonin reuptake inhibitors/serotonin norepinephrine reuptake inhibitors) or referral to a clinical psychologist for cognitive behavioural therapy (Figure 3) [1, 6, 14, 47]. In patients presenting with a symptom complex of reflux and early satiety, abdominal distension, nausea, vomiting, or postprandial abdominal pain, gastroparesis should be considered. This can be confirmed by exclusion of mechanical obstruction and gastric-emptying technetium scintigraphy [14, 15].

## GORD: tailoring interventions

Patients with disease progression or persistent GORD symptoms despite optimal PPI therapy, those who fail deprescribing interventions, or those with GORD who are considered for interventional procedures should be assessed by endoscopy/mucosal biopsy and ambulatory 24-hour combined pH impedance–high-resolution manometry (HRM) [1, 6, 10, 13–15, 47].

### Endoscopy and pH monitoring

Endoscopic assessment includes LA grading of erosive oesophagitis (A–D), Hill grading of diaphragmatic hiatus flap valve, measurement of axial length of hiatus hernia, Prague classification, and biopsy of Barrett’s oesophagus when present. Two to four biopsies from the distal and proximal oesophagus (off PPI treatment) should be collected for histopathological assessment for eosinophilic oesophagitis. Ambulatory 24-hour multichannel intraluminal impedance pH (MII-pH) monitoring can be used to detect all types of reflux, including acid, weak acid, or weak alkaline reflux, as well as the number of reflux events, with >80 reflux events in 24 hours considered abnormal and <40/24 hours considered normal. Prolonged pH monitoring (48–96 hours) with wireless capsule telemetry has the advantages of improved tolerance, symptom recording, and diagnostic yield, but requires endoscopy for placement and does not allow oesophageal manometry or measurement of weak acid reflux (pH 4–7). Weak acid reflux may still produce symptoms and signs of oesophageal mucosal injury in some patients due to the inflammatory effects of pepsins and bile acids; other patients may experience symptoms of volume regurgitation or reflux hypersensitivity. Bilirubin absorbance monitoring can be useful in diagnosing duodeno-oesophageal reflux, which usually responds poorly to PPI therapy [1, 6, 10, 13–15, 47].

The presence of LA Grade B oesophagitis or higher on endoscopy, or >6% oesophageal acid exposure time (AET) on 24-hour pH monitoring confirms a GORD diagnosis (Lyon consensus). The DeMeester reflux scoring system is based on a composite score of six parameters: longest duration of reflux, total number, number >5 min and overall percentage time of acid reflux events (oesophageal pH < 4), and percentage of reflux events in supine or upright positions. A DeMeester score of >14.72 indicates gastro-oesophageal acid reflux and a score of >100 severe acid reflux [1, 6, 10, 13–15, 47].

### Exclusion of alternative diagnoses

Endoscopy, combined ambulatory 24-hour MII-pH and HRM, and symptom association probability (SAP)/symptom index (SI) are used to identify those patients with:

- non-pathological acid reflux (AET < 4%);
- disorders of OGJ outflow (achalasia subtypes I–III or OGJ outflow obstruction);
- major oesophageal motility disorders (distal oesophageal spasm, hypercontractile oesophagus, absent oesophageal contractility); and
- functional disorders (rumination, reflux hypersensitivity, functional heartburn, supragastric belching).

This enables triaging of patients with true GORD potentially suitable for anti-reflux procedures and those with pathologies requiring different therapies [47].

### HRM

HRM is used to document decreased resting LOS pressure (<10 mmHg), failure of LOS relaxation (integrated relaxation pressure > 15 mmHg), hiatus hernia morphology and IOM, and position trans-nasal catheters for 24-hour pH monitoring. According to the Chicago classification version 3.0, IOM is defined as a distal contractile integral (DCI) value of <450 mmHg·s·cm in 50% or more of liquid swallows on HRM. Hypercontractile oesophageal peristalsis is characterized by DCI value of ≥8,000 mmHg·s·cm, failed oesophageal peristalsis by DCI value of <100 mmHg·s·cm, and normal peristalsis by DCI value of 450–8,000 mmHg·s·cm [15].

When a patient is on PPI treatment, the HRM post-reflux swallow-induced peristaltic wave (PSPW) index can clearly distinguish between true NERD and functional heartburn. In patients with borderline GORD (AET: 4%–6%), abnormal nocturnal baseline impedance and an abnormal PSPW index occur more frequently than in patients with functional heartburn. An abnormal response to multiple rapid swallows on preoperative HRM can indicate impaired oesophageal body contractile reserve and predict post-operative dysphagia after laparoscopic fundoplication. Multiple rapid swallows may also identify patients with motility disorders not found on manometry alone, including variants of achalasia or distal oesophageal spasm. Other provocation tests that can improve the sensitivity and specificity of HRM in diagnosing motility disorders include rapid drink challenge and apple viscous swallow tests [15].

Between 1% and 3% of patients with refractory GORD symptoms have an underlying diagnosis of achalasia, managed with per-oral endoscopic myotomy or laparoscopic Heller’s myotomy with fundoplication. Absent oesophageal peristalsis with normal LOS relaxation is found in 3% of oesophageal manometry tests for GORD, often associated with scleroderma oesophagus. The “tailoring” of anti-reflux surgery to Nissen fundoplication in patients with GORD and normal oesophageal motility and partial fundoplication in the presence of oesophageal dysmotility is debated [1]. After excluding histological evidence of reflux-associated mucosal injury by endoscopy, pathological acid exposure by MII-pH monitoring, and motility disorders by HRM, a positive heartburn SAP or SI (SI ≥ 50%, SAP > 95%) for reflux episodes indicates reflux hypersensitivity and a negative heartburn SAP/SI (SI < 50%, SAP ≤ 95%) indicates functional heartburn (Figure 3) [1, 6, 10, 13–15, 47].

### Truly refractory GORD

Thorough assessment is important, with one study reporting that heartburn symptoms are truly refractory and acid reflux-related in only 21% of patients [1]. Among the remaining patients, 12% responded to twice-daily omeprazole when administered under strict instructions, 6% had non-GORD diseases (eosinophilic oesophagitis, achalasia), 27% had functional heartburn, and 15% were excluded for miscellaneous reasons [1]. Treatment success (defined as >50% improvement in GORD–HRQL scores) in the 21% of patients identified to have truly refractory GORD was more often achieved in those randomized to receive laparoscopic Nissen fundoplication (67%) compared with those randomized to active medical treatment (28%, *P *=* *0.007) or placebo medical treatment (11.5%, *P *<* *0.001) [1, 47]. In patients with extra-oesophageal symptoms refractory to PPI therapy, and when there is a lack of objective evidence of pathological reflux, invasive endoscopic or surgical GORD interventions should be avoided [1].

## GORD: procedural intervention vs PPI treatment

Despite PPIs being the current first-line treatment for GORD, up to 40% of patients are dissatisfied with their medical management. Although PPIs may effectively treat acid reflux, they may not improve the symptoms of regurgitation. Progression of GORD with maximal medical treatment also occurs in as many as 45% of patients [49]. Among patients with Grade C oesophagitis, 100% relapse within 6 months of PPI cessation [1]. Without procedural intervention, continuous, often lifelong therapy is required for such patients [5].

### Laparoscopic fundoplication vs PPIs

The 2015 Cochrane review compared four selected RCTs of laparoscopic fundoplication (LF) vs long-term treatment with PPIs. This encompassed 1,160 patients with GORD followed for 1–5 years [50]. Respective outcomes were:

- better short-term (<12 months) GORD–HRQL with LF (standard mean difference [SMD]: 0.58, 95% CI, 0.46–0.70);
- less medium-term (1–5 years) heartburn with LF (4.2% vs 22.2%, RR: 0.19, 95% CI, 0.10–0.34);
- less medium-term reflux symptoms with LF (2.1% vs 13.9%; RR: 0.15, 95% CI, 0.06–0.35);
- increased serious adverse events (SAEs) in 18.1% (60/331) of surgical patients vs 12.4% (38/306) of medical patients (RR: 1.46, 95% CI, 1.01–2.11; pooled studies* *=* *2);
- higher rates of medium-term dysphagia in 10.1% (29/288) of surgical patients vs 1.9% (5/266) of medical patients (RR: 5.36, 95% CI, 2.1–13.64; studies* *=* *1); and
- no difference in long-term (>5 years) dysphagia rates (RR: 0.90, 95% CI, 0.57–1.42; participants* *=* *228, studies* *=* *1).

The Cochrane review cited problems with the available studies including performance, detection, attrition, and reporting bias. The authors called for further RCTs of LF vs PPI therapy to be conducted with outcome assessor blinding, including long-term follow-up of patient-oriented outcomes [50].

Other systematic reviews and long-term RCTs have demonstrated equivalent or superior outcomes for LF vs PPI treatment [51–54]. For example, the 2021 meta-analysis by Mckinley *et al.* [52] included 20 RCTs and 13 cohort studies of surgical fundoplication vs medical management. Four pooled RCTs showed that LF resulted in better normalization of oesophageal pH than PPIs (mean difference: 2.11, 95% CI, 1.83–2.38, *I*^**2**^* *=* *0%). One RCT showed superior improvement in DeMeester scores with surgery (mean difference: 9.0, 95% CI, 3.25–14.95). Five pooled RCTs reported long-term (>5 years) symptom control and LF was found to be superior to PPI treatment (RR: 0.79, 95% CI, 0.63–0.999, *I*^**2**^* *=* *87%), although there was heterogeneity among the studies. Short-term (<5 years) HRQL scores were worse with PPIs (SMD: −0.51, 95% CI, −0.63 to −0.40, *I*^**2**^= 0%) in four RCTs with low heterogeneity and a total of 575 medical patients and 594 surgical patients. Long-term HRQL scores were equivalent in three RCTs involving 282 patients treated with PPIs and 227 patients treated with LF (SMD: 0, 95% CI, −0.23 to 0.23, *I*^**2**^* *=* *35%). In three pooled RCTs, long-term use of PPIs after surgery was observed in 86 of 308 patients (28%) randomized to LF. Long-term dysphagia rates were compared in five RCTs involving a pooled total of 628 patients treated medically and 600 treated with surgical fundoplication. There was no statistically significant difference between the groups (RR: 0.92, 95% CI, 0.50–1.67, *I*^**2**^* *=* *61%), although heterogeneity was found. Long-term gas-bloat syndrome was significantly less likely in medically managed patients (RR: 0.70, 95% CI, 0.55–0.89) [52].

### Endoscopic vs laparoscopic GORD interventions

Only a small percentage of suitable GORD patients are ever referred for surgical management. This is related to referral patterns, perceived and actual risks of LF, the efficacy of PPIs, and the rates of reflux recurrence and recommencement of PPIs after LF [5, 56]. LF is the standard surgical procedure for proven GORD that has failed medical management or for severe or complicated GORD [52]. Other less invasive interventions, including endoscopic radiofrequency ablation (RFA), endoscopic transoral incisionless fundoplication (TIF), and laparoscopic magnetic sphincter augmentation (MSA), have been developed as potential alternatives to LF or lifelong PPI treatment. RFA (Stretta^®^) involves the application of radiofrequency energy to create a thermal injury to the muscularis propria and submucosa below, at, and above the LOS. This is thought to cause muscular hypertrophy and submucosal fibrosis, which decrease TLOSR and increase LOS yield pressure. TIF constructs an endoscopic 200°–270° full-thickness fundoplication with a high-pressure zone 2–3 cm above the OGJ, similar to Toupet fundoplication. The MSA or LINX^®^ procedure involves laparoscopic placement around the distal oesophagus of an expandable collar of titanium beads with magnetic cores of neodymium linked by a flexible titanium wire. This is designed to bolster the LOS, but also allow swallowing, belching, or vomiting [1, 49].

**Figure 4.**
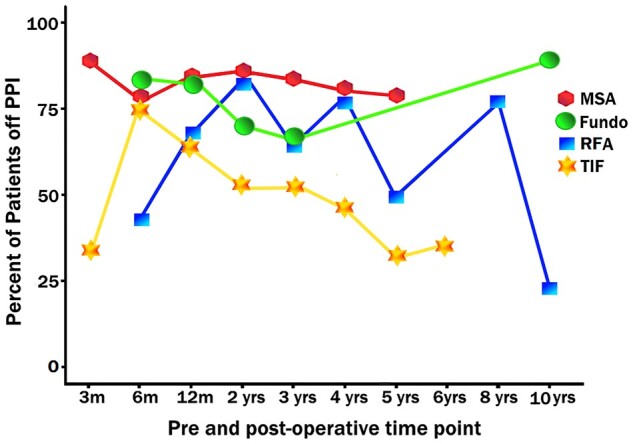
Weighted means for percentage of patients off PPI therapy after MSA, RFA, TIF, and fundoplication [49]. MSA, magnetic sphincter augmentation; PPI, proton-pump inhibitor; RFA, radiofrequency ablation; TIF, transoral incisionless fundoplication. In long-term follow-up (5 years), both TIF and RFA had progressive failure rates for keeping patients off PPIs, as compared with both fundoplication and MSA.

A 2021 systematic review assessed four common end points after invasive GORD interventions: the percentage of patients off PPIs, GORD–HRQL score, DeMeester score, and AET during 24-hour pH manometry [49]. At 1 year post-procedure, 85% of patients who underwent MSA were off PPIs, followed by LF (84%), RFA (62%), and TIF (61%). All four procedural interventions achieved a >50% improvement in GORD–HRQL scores at 12 months. LF produced the greatest effect (87.9% improvement in GORD–HRQL score), followed in descending order by MSA (86.6%), TIF (76.4%), RFA (68%), and PPI (49.8%). Normalization of weighted mean oesophageal AET (<4%) and DeMeester scores (<14.72) was achieved by LF and MSA but not by TIF or RFA at 12 months (Table 1) [49]. In long-term follow-up at 5 years, both TIF and RFA procedures progressively failed to keep patients off PPIs as compared with both LF and MSA (Figure 4) [49].

**Table 1.** Comparison of gastro-oesophageal reflux disease (GORD) treatments for laparoscopic fundoplication (LF), magnetic sphincter augmentation (MSA), transoral incisionless fundoplication (TIF), radiofrequency ablation (RFA), and proton-pump inhibitor (PPI) prior to and 12 months after treatment

GORD treatment	AET % pH < 4		DeMeester score		GORD–HRQL			Percentage of patients off PPIs at 12 months post-treatment
	Prior	12 months	Prior	12 months	Prior	12 months	Percentage decrease	
LF	11.41	1.27	35.69	10.18	23.1	2.8	87.9%	84%
MSA (LINX^®^)	10.16	3.24	32.06	13.01	23.3	3.1	86.6%	85%
TIF (EsophyX^®^)	9.78	7.08	30.40	23.03	25.9	6.11	76.4%	61%
RFA (Stretta^®^)	11.16	7.57	41.72	30.46	22.6	7.23	68.0%	62%
PPI	9.33	4.29	32.53	28.23^a^	23.9	12.0	49.8%	0

Mean weighted objective and subjective parameters included oesophageal acid exposure time (AET) represented as percentage of time with pH of <4 on pH monitoring (normal range: <4%), combined DeMeester score (normal range: <14.72), gastro-oesophageal reflux disease health-related quality-of-life score (GORD–HRQL treatment success defined as >50% decrease), and percentage of patients not taking PPIs at 12 months post-treatment [49].

a Data only available for 6 months’ treatment duration.

MSA has emerged as the most effective alternative treatment to LF in selected patients with GORD. MSA was shown to be clearly superior to twice-daily PPI therapy in a RCT involving patients with moderate to severe regurgitation who had already been treated with once-daily PPIs for 8 weeks. Although there are no published RCTs, observational cohort studies and meta-analyses have reported equivalent symptom control and GORD-specific HRQL, lower rates of gas-bloat syndrome (OR: 0.34; 95% CI, 0.16–0.71), and shorter operative time and length of hospital stay with MSA as compared with LF [1, 10].

No RCTs have directly compared LF and TIF, and systematic reviews report the inconsistent clinical effectiveness of TIF [49, 57–60], which has influenced GORD management algorithms [1, 57]. The most recent systematic review and meta-analysis published in 2021 concluded that TIF was a safe and effective alternative to LF, particularly in those patients with small (<2.5 cm) or no hiatus hernia, were intolerant of PPIs, who refused lifelong PPIs or surgery, or were at risk of post-surgical adverse events [60].

Other endoscopic interventions for GORD include Medigus Ultrasonic Surgical Endostapler (MUSE^®^), endoscopic full-thickness plication (GERDx^®^), and anti-reflux mucosal interventions (ARMI). ARMI procedures include anti-reflux mucosectomy (ARMS) using endoscopic submucosal dissection or mucosal cap/snare of gastric cardia mucosa, anti-reflux mucosal ablation (ARMA) with argon plasma coagulation, and anti-reflux band ligation (ARBL). Of these, only the ARBL procedure has been investigated in a clinical RCT; however, this did not satisfy the Consolidated Standards of Reporting Trials quality requirements [57]. At present, these procedures should be considered experimental techniques until larger prospective studies/RCTs have been completed. They have varying clinical success (43%–100%), complication rates (0–17%), and levels of difficulty. The ARMI techniques are quicker to perform and require less complex endoscopic equipment and anaesthesia. SAEs have mainly been observed with plication/stapling techniques, with Clavien–Dindo Grade III/IV complications including dysphagia, pneumothorax, perforation, bleeding, mediastinal abscess, and thoracic empyema being similar to those reported with TIF. The risk of SAEs in a pooled series of MUSE procedures was found to be 3.5% and 2.4% for TIF [1, 57]. The pooled perforation rate with ARMI was 2.4% and zero with ARMA or ARBL. A double-blind RCT to assess clinical success and safety of ARMA is currently being conducted [57]. The relatively low SAE incidence and utility of the three ARMI techniques are promising, together with significant improvements in GORD–HRQL, DeMeester scores, oesophageal AET, LOS resting pressure, total reflux episodes, and PPI dependence in short-term (6 months) follow-up. Limited medium-term follow-up (1–3 years) exists for ARMS and ARMA. Objective measurements of long-term durability and efficacy and sham/placebo comparators are required to confirm the place of ARMI in treating GORD [6, 57, 61].

### Nissen vs partial fundoplication

By reconstructing the anatomical components of the anti-reflux barrier, LF is effective in treating patients with PPI-responsive GORD and selected patients with PPI-refractory GORD. Reported control of heartburn and regurgitation is 90% at 10-year follow-up and 80% at 20-year follow-up [13]. Whilst Nissen fundoplication (360° wrap) is the most frequently performed procedure and considered by some to be the gold standard, partial fundoplication (Toupet-Posterior [270°], Dor-Anterior [180°], Watson-Anterior [90°]) is designed to minimize post-operative dysphagia and gas-bloat syndrome (postprandial fullness, bloating, inability to belch, epigastric pain, and flatulence) [52, 62–64]. The respective rates of dysphagia and gas bloat 6–12 months after Toupet (3% and 23%) are lower than after Nissen fundoplication (7% and 36%). This is offset by a higher rate of short-term complications after Toupet fundoplication (bleeding/perforation). When Nissen fundoplication was performed with a floppy wrap without oesophageal fixation, the dysphagia rates were similar to those for Toupet fundoplication [13]. The differences in side effects between the Nissen and Toupet procedures appear to equalize at 10-year follow-up [64]. Dor fundoplication is associated with higher acid reflux events, lower LOS resting pressure on pH manometry, and increased requirement for PPIs (29% vs 8%, *P *=* *0.008), but this is offset by less dysphagia as compared with Nissen fundoplication in long-term RCT follow-up (12–14 years) [64]. The Society of American Gastrointestinal and Endoscopic Surgeons (SAGES) 2021 guidelines suggested either partial or 360° fundoplication could be used for the treatment of GORD, with the choice of technique related to the requirement for control of reflux symptoms vs potential risk of post-operative dysphagia [65].

### Recurrent or persistent symptoms after laparoscopic GORD intervention

Acute dysphagia after LF/hiatus hernia repair can be experienced in up to 50% of patients due to localized post-operative oedema and inflammation, but usually resolves within 3 months [55]. Persistent dysphagia (>3 months) is more commonly related to functional or anatomical abnormalities of the OGJ, rather than oesophageal dysmotility. This includes length, type, tightness, and integrity of the fundoplication and the nature of the crural repair. Endoscopy and solid/liquid phase barium swallow fluoroscopy are useful initial investigations. These can demonstrate proximal migration of the fundoplication, slipped fundoplication, hiatal stenosis, or impaired passage of a swallowed liquid or solid bolus. HRM can identify new-onset OGJ outflow obstruction (particularly on solid swallows) or oesophageal dysmotility. HRM can predict the response (58%) to oesophageal dilatation, with non-responders requiring revisional surgery. Patients with new-onset dysphagia post-LF have elevated median integrated relaxation pressure and inadequate deglutitive relaxation of the OGJ neo-high-pressure zone as compared with asymptomatic post-LF patients. The OGJ neo-high-pressure zone is a combination of the phreno-oesophageal ligament, crural diaphragm, LOS, and fundoplication. A proportion of patients (29%) with post-LF dysphagia will have normal HRM, with structural problems identified at endoscopy or barium swallow. Some patients will experience persistent post-operative dysphagia despite having a technically sound Nissen LF [61].

Between 2% and 12% of patients undergo oesophageal dilatation after fundoplication, with procedural success more likely in patients with intact fundoplication and normal oesophageal peristalsis [66, 67]. An analysis of two pooled RCTs of post-operative outcomes revealed that 5 of 119 patients (4%) who had partial fundoplication required endoscopic dilatation for post-operative dysphagia, which was not statistically different to the dilatation requirement after Nissen fundoplication in 3 of 110 patients (3%) [52]. In patients with holdup of a solid barium bolus after Nissen fundoplication, conversion to a partial fundoplication should be considered if there is no improvement with oesophageal dilatation.

To prevent post-operative dysphagia after LF, it is important to construct the posterior crural repair so that the distal oesophagus is not pushed anteriorly and adequate space remains for food bolus expansion of the distal oesophagus. Routine intraoperative use of a 50–56 Fr oesophageal bougie to calibrate the hiatal closure and fundoplication is advocated by some but avoided by others. This is due to conflicting evidence for the benefit of bougie insertion, risk of bougie oesophageal perforation (1%), and application of a standardized surgical technique during primary LF [13, 55, 64, 65, 67]. Patients with GORD and preoperative dysphagia are more likely to experience dysphagia after LF and should be carefully assessed and counselled prior to intervention [61, 67]. Preoperative oesophageal hypomotility is not a consistent risk factor for post-Nissen LF dysphagia, except in those patients with absent peristalsis [61].

Recurrent or persistent reflux symptoms after LF can be related to initial patient selection, inadequate preoperative workup, misdiagnosis, total vs partial fundoplication, inadequate intraoperative oesophageal mobilization, technical problems with the crural repair or construction of the fundoplication, hiatus hernia recurrence, and patient factors [6, 10, 52, 68]. Analysis of a Swedish population-based registry of 2,655 patients who underwent primary LF between 2005 and 2014 showed recurrent reflux in 17.7% of patients (470 of 2,655) at 5 years’ mean follow-up. Significant patient risk factors included older age, female sex, and patient co-morbidity. The majority of these patients (393/470) were managed with PPI/H2RA therapy and 77/470 patients had revisional surgery [68]. Recommencement of PPIs after Nissen LF may not be a reliable indicator of failed surgical treatment, as the majority of patients (76%) with post-operative symptoms do not have significant acid exposure on 24-hour pH monitoring. Those patients who do have objective evidence of oesophageal acid exposure have a 53-fold increased risk of a disrupted, abnormally placed fundoplication requiring revision [69]. In a Danish population-based cohort study of 4,258 anti-reflux operations between 2000 and 2017, the frequency of revisional surgery increased with time: 3.1% at 1 year and up to 12.8% at 15 years after primary LF. This included surgery for recurrent hiatus hernia [70]. In an Australian study of LF, revisional surgery was required in 5.6% of patients (98 of 1,751) at a median time of 26 months (range 2–143 months) after primary LF. In this study, the indications for reoperation included dysphagia (48%), proven recurrent reflux (33%), para-oesophageal hernia (15%), and atypical symptoms (4%) [71].

It is critical to properly reassess individual patients who have recurrent reflux, regurgitation, or chest pain after LF, as this will determine whether they can be surgically or medically managed [69, 72]. Assessment should include endoscopy, 24-hour pH manometry, and swallowing MRI/barium fluoroscopy. Endoscopic assessment should ideally be performed by an experienced foregut surgeon or oesophagologist, as there is significant interobserver variation in the recognition of a disrupted wrap or recurrent hiatus hernia [73]. A substantial number of patients with normal AET on pH monitoring but persistent symptoms after LF have alternative diagnoses including gallstones, irritable bowel syndrome, *H. pylori*-related PUD, coronary artery disease, or psychiatric disorder [72]. Swallowing MRI is able to identify fundoplication disruption, slippage, recurrent hiatal hernia, and oesophageal motility disorders. It has good interobserver reliability even with inexperienced readers and may provide a suitable alternative to barium fluoroscopy [74].

Outcomes can be less satisfactory after revisional surgery than primary LF. Up to 10% of patients will report persistent reflux or dysphagia after revisional surgery. Respective 30- and 90-day complication rates are also higher after revisional surgery (7.0% and 8.3%) compared with primary LF (3.4% and 4.8%) [70]. Reoperative surgery can be technically challenging, associated with longer operating times and more frequent open conversions than initial LF (8% vs 0%) [71]. Patients with morbid obesity and recurrent reflux may benefit from laparoscopic Roux-en-Y gastric bypass surgery (RYGB) rather than fundoplication revision, as RYGB will divert gastric acid or bile away from the oesophagus and also effectively treat obesity [1, 75].

Intervention after MSA placement is infrequently required. This is again dependent upon careful patient selection and should be performed according to the manufacturer’s exclusion criteria: hiatus hernia of >3 cm, LA Grade C or D oesophagitis, IOM, BMI of >35 kg/m^2^, or previous anti-reflux surgery. Post-operative dysphagia is frequent (43%–83%) after MSA but tends to resolve after 3 months. The incidence of persistent dysphagia varies between 3% and 19% in different studies [76–79]. Risk factors include preoperative dysphagia, severe preoperative IOM, intact hiatus, or smaller MSA device size. The majority of patients who undergo oesophageal dilatation for dysphagia after MSA have favourable responses (67%–76.9%) [76]. In a global study, MSA was reported to have an erosion rate of 0.3% (29 of 9,453 implants) at 4 years post-implantation [77]. The most common symptom of erosion was new-onset dysphagia. Device removal for erosion was achieved by an initial endoscopic approach for the intraluminal portion followed by a laparoscopic approach. Between 3.0% and 9.2% of MSA devices require explantation due to dysphagia, persistent or recurrent GORD symptoms, vomiting, chest pain, or device erosion. Subsequent procedures performed after MSA removal include repeat MSA insertion (33%), fundoplication (21%), gastrectomy (4%), or no additional procedure (42%) [77–79].

## GORD management: expert guidelines

There are some differences between expert consensus recommendations with regard to indications for LF. The 2008 American Gastroenterology Association (AGA) Position Statement recommended LF for proven GORD poorly controlled by PPIs (especially regurgitation), patients intolerant of PPI therapy, or when anti-reflux surgery offered similar efficacy to PPI treatment. The AGA recommended against anti-reflux surgery in patients with GORD that is well controlled under medical management [80]. The recent 2022 AGA Best Practice update recommends a tailored approach to the diagnosis and management of GORD, with the severe oesophagitis phenotype (LA Grade C or D, AET > 12%, DeMeester score > 50 or bipositional reflux) requiring long-term continuous PPI treatment or invasive anti-reflux procedures [81]. This update suggests that LF and MSA are effective treatments for patients with proven GORD and TIF is an effective endoscopic treatment option in carefully selected patients. Specified criteria for invasive anti-reflux procedures included confirmatory evidence of pathological GORD, assessment of oesophageal peristaltic function, and exclusion of achalasia [81].

The ACG 2022 Clinical Guidelines recommend LF for long-term management in patients with objective evidence of GORD, identifying those with LA Grade C or D oesophagitis, large hiatus hernia, and/or persistent, troublesome GORD as most likely to benefit from surgery. The ACG suggests anti-reflux surgery be considered for PPI-refractory GORD with documented objective GORD evidence and regurgitation as the primary symptom. MSA could be considered as an alternative to LF in patients with regurgitation who have failed medical management. The use of RFA as an alternative to medical or surgical anti-reflux therapies is not supported in the ACG guidelines. However, the use of TIF can be considered in patients who do not have a hiatus hernia of >2 cm, do not have LA Grade C or D oesophagitis, or do not want to undergo anti-reflux surgery [1]. The SAGES 2021 guidelines suggest that patients with confirmed chronic or chronic refractory gastro-oesophageal reflux be managed with surgical fundoplication rather than continued medical treatment [65], while the UK National Institute for Health and Care Excellence (NICE) 2019 guidelines suggest LF in patients with a confirmed diagnosis of acid reflux and adequate symptom control with PPI therapy but who do not wish to continue PPIs or cannot tolerate PPIs (Table 2) [48].

**Table 2.** Indications for laparoscopic anti-reflux surgery (LF) [1, 48, 65, 80, 81]

Guidelines	Indications
AGA	Patients with oesophagitis who are intolerant of PPI therapy
	Patients with symptoms of oesophageal GORD syndrome poorly controlled by PPI therapy, especially in the setting of persistent troublesome regurgitation, or patients with severe oesophagitis phenotype. Confirmatory evidence of pathological GORD, assessment of oesophageal peristaltic function and exclusion of achalasia is required before invasive anti-reflux procedures are performed
	Carefully selected patients with extra-oesophageal GORD syndromes in whom a reflux causality has been established to the greatest degree possible
SAGES	Patients with chronic GORD who opt for LF despite successful medical management
	Patients who have chronic refractory GORD or failed medical management (inadequate symptom control, severe regurgitation not controlled with acid suppression, or medication side effects)
	Patients who have GORD complications (Barrett’s oesophagus, peptic stricture)
	Patients who have extra-oesophageal manifestations (aspiration, asthma, chest pain, cough, hoarseness) with proven GORD on pH/impedance testing
ACG	Patients with objective evidence of GORD are recommended to have anti-reflux surgery, performed by an experienced surgeon, for long-term therapy. Those who have severe reflux esophagitis (LA Grade C or D), large hiatal hernia, and/or persistent, troublesome GERD symptoms are likely to benefit most from surgery
	Patients who have regurgitation as their main PPI-refractory symptom with abnormal GOR documented by objective testing can be considered for anti-reflux surgery, MSA, or TIF
	Patients treated for extraesophageal reflux disease with objective evidence of reflux can be considered for surgical or endoscopic anti-reflux procedures
NICE	Patients with a confirmed diagnosis of acid reflux and adequate symptom control with PPI therapy, but who did not wish to continue PPIs or cannot tolerate PPIs

ACG, American College of Gastroenterology; AGA, American Gastroenterological Association; GOR, gastro-oesophageal reflux; GORD, gastro-oesophageal reflux disease; LF, laparoscopic fundoplication; MSA, magnetic sphincter augmentation; NICE, UK National Institute for Health and Care Excellence; PPI, proton-pump inhibitor; SAGES, Society of American Gastrointestinal Endoscopic Surgeons; TIF, transoral incisionless fundoplication.

## GORD treatment: cost-effectiveness

Many national and societal GORD treatment consensus guidelines do not include long-term treatment costs or cost-effectiveness analysis.

### Break-even costs of LF vs PPI

**Figure 5.**
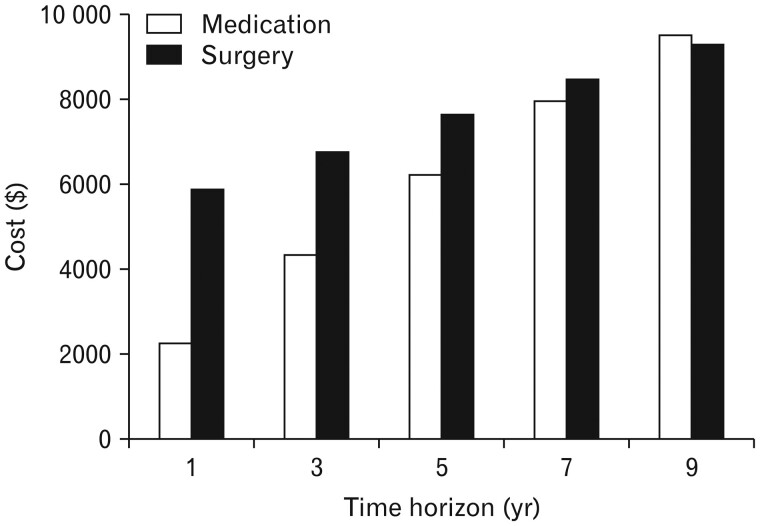
One-way sensitivity analysis according to various time horizons [83]. Cost comparison model (US$) between PPI and laparoscopic anti-reflux surgery in a patient aged 50 years old with severe GORD who required a continuous double dose of PPIs. The overall time horizon was 10 years and all estimates were discounted at 5% per year. Laparoscopic anti-reflux surgery became less expensive than continuous PPIs after 9 years. Reprinted from *J Neurogastroenterol Motil* 2020;**26**:215–23 with permission.

GORD treatment cost analysis suggests an equivalence between LF and 5–10 years’ continuous supply of high-dose PPIs (Figure 5) [82–84]. The in-hospital cost of Nissen LF surgery influences the break-even time period more than PPI recommencement after Nissen LF, Nissen LF reoperation rates, PPI relapse rates, future PPI prices, PPI dose escalation, or discount rates [81]. This is important when comparing GORD treatment costs between different countries and national health systems, and when designing models for decision analysis [80]. Even after factoring in the proportion of patients who may restart PPIs after LF, surgical intervention with LF is still associated with lower long-term costs and better patient satisfaction. Long-term cost-effectiveness and symptom control should be considered when managing GORD, particularly in younger patients who face the prospect of lifelong PPI treatment [1, 2, 5, 53, 54, 56, 85, 86].

### Markov models of treatment cost-effectiveness

**Figure 6.**
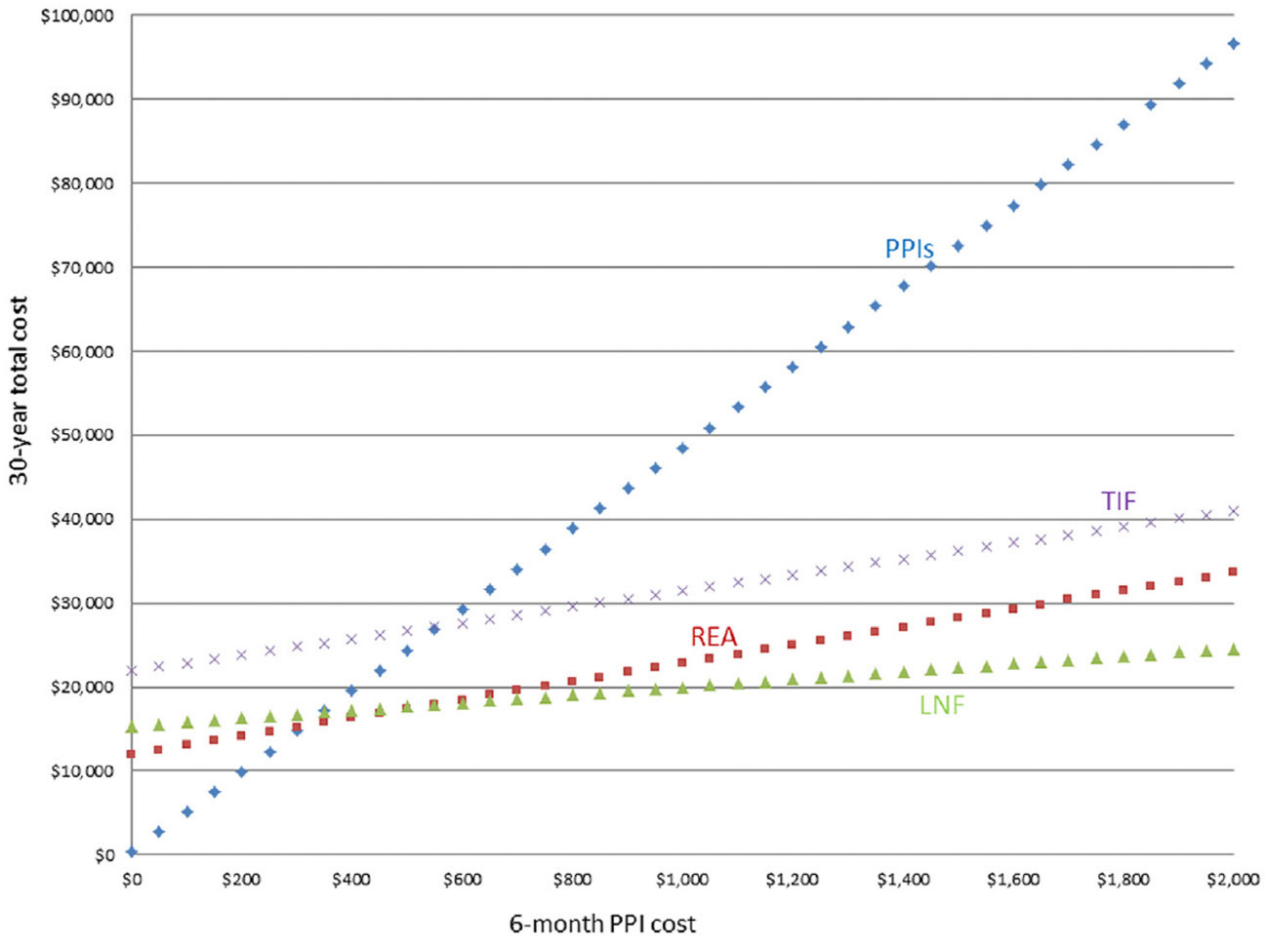
Thirty-year cost comparison (US$) between each strategy at varying PPI costs [56]. In the base model at a 6-monthly over-the-counter generic omeprazole 20 mg bid cost of US$204, PPI therapy was the least expensive option, but REA and LNF were the most cost-effective treatment options over 30 years. When the PPI cost exceeded US$540/6 months, Nissen fundoplication became the most cost-effective treatment. PPI, proton-pump inhibitor; LNF, laparoscopic Nissen fundoplication; REA (Stretta^®^), radiofrequency energy application to the lower oesophageal sphincter; TIF (EsophyX^®^), transoral incisionless fundoplication.

A 10-year Markov model published in 2003 suggested that up to 50% of patients with GORD well controlled with PPIs would benefit from LF [87]. In 2015, cost-effectiveness (cost per quality-adjusted life-year gained) of Nissen LF, TIF 2.0, Stretta^®^, and chronic PPI therapy were compared in a more contemporary Markov model on a 30-year time horizon [56]. The base case was a 45-year-old male patient with symptomatic GORD taking 20 mg twice-daily of omeprazole. The model parameters included treatment success rates, PPI dose escalation, relapse to PPIs after endoscopic therapy, requirement for Nissen LF, relapse to PPI after Nissen LF, and revisional LF. In the base model using a 6-month over-the-counter twice-daily generic-omeprazole cost of US$204 (US$34/month), PPI therapy was the least expensive but also the least effective treatment option. Stretta^®^ and laparoscopic Nissen LF were the most cost-effective GORD treatment options (US$3,417.53 and US$6,119.63 per quality-adjusted life-year gained, respectively) over 30 years. When the PPI cost exceeded US$540/6 months (US$90/month), Nissen LF became the most cost-effective treatment at 5, 10, and 30 years. The increasing cost of PPIs was closely related to the use of prescription and/or brand-name PPIs. Nissen LF dominated TIF at all time points due to TIF being a more expensive and less effective treatment (Figure 6). It was concluded that in patients who require high-dose or expensive PPIs, early referral for procedural GORD treatment should be considered [56].

### SAGES meta-analysis and cost-effectiveness

The 2021 meta-analysis used to update the SAGES GORD management guidelines found LF to be more expensive than PPI therapy based on up-front raw costs [52]. Laparoscopic fundoplication was, however, cost-effective in three of five studies comparing LF to long-term medical management, with greater quality-of-life improvement after LF [52]. When compared to LF, robotic fundoplication was found to have similar safety and efficacy but was more expensive in terms of perioperative, inpatient, in-hospital, and yearly investment and maintenance costs [52, 65].

### MSA vs LF costs

A recent analysis showed no significant differences in the safety, symptom control, and total in-hospital costs of MSA (US$48,491) compared with Nissen LF (US$50,111) in patients treated for uncomplicated GORD. This included hiatus hernia repair in both groups as required during surgery. MSA had a shorter procedural time (66 vs 82 min, *P *<* *0.01) and overall hospital stay (17 vs 38 hours, *P *<* *0.01) than LF. MSA was also associated with fewer laboratory tests, less medication (usage of narcotics and anti-emetics), and decreased charges for operating theatre services, hospital room, and board. This offset the MSA device billing cost (∼US$5,000) [88]. In a large healthcare system cost analysis, MSA was associated with a 66% reduction in disease-related reimbursement costs as compared with a 46% reduction with Nissen LF at 12 months post-surgery (*P *=* *0.0001) [89]. However, the study did not factor in future costs of MSA device explantation or LF revision. It also used insurance reimbursement costs for PPIs rather than over-the-counter costs [89]. In another study, modelling predicted that introduction of MSA into current practice would potentially improve the budgetary impact of treating PPI-refractory GORD for commercial payers in the USA [90].

## Conclusions

The most effective medical treatment for proven GORD is PPI therapy. The benefits of potent gastric acid suppression, healing of erosive oesophagitis, and GORD symptom control outweigh the potential side effects of PPIs. However, there is widespread inappropriate prescribing of PPIs with respect to dosage, indication, and duration in many Western and Eastern nations. This high prevalence of PPI use is associated with considerable economic costs to national health services and can make otherwise infrequent PPI adverse events clinically relevant. PPI prescribing was restricted by the Australian PBS in 2019 in an attempt to reduce subsidized drug costs, particularly for esomeprazole. The broad introduction of PPI stewardship in hospitals would be an effective intervention for preventing the initiation, continuation, and perpetuation of unnecessary PPI use.

Treatment guidelines for GORD suggest a short-term course of high-dose PPI medication. After the successful resolution of pathology or symptoms, deprescribing of PPIs should be attempted when appropriate. Prescribing of long-term PPIs should be avoided when GORD is unproven. LF for GORD is indicated as being clinically successful and cost-effective in suitable patients with comprehensive preoperative evaluation as compared with long-term PPI treatment. LF in long-term management of PPI-responsive GORD is supported by SAGES, NICE, and the ACG, and PPI-refractory GORD by the AGA and SAGES. The importance of establishing a definitive diagnosis prior to invasive management is emphasized, especially in PPI-refractory heartburn. Alternative laparoscopic (MSA) and endoscopic techniques (TIF, ARMI) may enable the tailoring of GORD management. However, further comparative studies are required. This paper has examined evidence-based guidelines for the prescribing and deprescribing of PPIs in primary care and hospital settings, and highlights the need for comprehensive education of involved health professionals, hospital staff, and patients. The aim of this narrative review was to compare the advantages and disadvantages of endoscopic, laparoscopic, and medical management of proven GORD. The findings of this review may assist in shared decision-making and choice of treatment for individual patients.

## Authors’ Contributions

T.L., J.T., P.T., and R.B.W. conceived and designed the project; T.L., J.T., P.T., and R.B.W. collected, analysed, and interpreted the data; T.L., J.T., and R.B.W. drafted the manuscript; T.L. and R.B.W. contributed equally to this paper. All authors read and approved the final version of the manuscript.
